# Repositioning Global Health: Decolonising Narratives and Practice in the African Context

**DOI:** 10.1002/puh2.70204

**Published:** 2026-02-27

**Authors:** Michael Sarfo, Ruth Owusuaa

**Affiliations:** ^1^ School of Human and Health Sciences University of Huddersfield Huddersfield UK; ^2^ Wiamoase SDA Hospital Wiamoase, Ashanti Region Ghana; ^3^ Tepa Nursing and Midwifery Training College Tepa, Ashanti Region Ghana

**Keywords:** Africa, decolonisation, global health

## Abstract

**Background:**

Global health practice in Africa remains shaped by enduring colonial legacies that privilege external expertise and undervalue indigenous knowledge systems. Despite widespread recognition of inequities in authorship, funding and representation, global health discourse often overlooks the deeper epistemic and cultural dimensions of decolonisation.

**Main Argument:**

This article argues that true decolonisation of global health requires dismantling the enduring mechanisms of coloniality, specifically epistemic injustice, misrepresentation and peripheral positioning, that continue to produce knowledge hierarchies and limit African autonomy. We propose a shift from a donor‐recipient logic to a partnership logic, where African institutions and scholars lead in defining priorities and generating knowledge. Examples such as the widespread use of herbal and traditional medicine, often the first line of care for rural communities, illustrate how local innovations are frequently dismissed as unscientific rather than recognised as valuable health assets. Furthermore, the routine portrayal of deprived African communities in global health imagery perpetuates stereotypes that reinforce dependency.

**Conclusion:**

Decolonising global health in Africa demands not only fair authorship and funding practices but also epistemic inclusion and representational justice. Global health must be repositioned to leverage Africa's own resources, cultural practices and institutional capacities to create contextually grounded, equitable and sustainable health systems.

## 1 Introduction

Global health is often framed as an endeavour to improve health outcomes worldwide, transcend national borders and leverage shared knowledge and resources. Yet as scholarship increasingly reveals, the field of global health remains deeply shaped by colonial legacies, power asymmetries and epistemic hierarchies. This persistence of coloniality, defined here as the persistent patterns of power, knowledge and resource control that exist beyond formal colonialism, means the dominant paradigm is driven largely by institutions, funders and researchers from the Global North [1, 2]. These actors often define priorities, methods, imagery and representation in ways that may reproduce dependency, marginalisation and misframing rather than genuine partnership. Previous studies have demonstrated how global health remains shaped by embedded coloniality, extractive knowledge flows and structural power asymmetries that privilege global north institutions [3, 4].

In the African context, the decolonisation of global health becomes both urgent and complex. On the one hand, many health interventions are designed elsewhere and transplanted into African settings, often with limited adaptation or community ownership. On the other hand, local knowledge systems, including traditional medicine, community‐based practices and cultural logics of care, are frequently dismissed, ignored or portrayed as inferior, rather than being engaged constructively [5].

For example, in many African countries, particularly among farming communities, traditional and herbal remedies remain central to managing health emergencies such as snakebites, a frequent occupational hazard. Studies from Kenya, South Africa and Gabon report that between 30% and 90% of snakebite victims first seek treatment from traditional healers or herbalists before reaching formal health [6, 7, 8]. This widespread reliance reflects not only limited access to antivenom and biomedical services but also deep trust in local knowledge systems that have evolved over generations. Yet, in global health narratives, such practices are often dismissed as unscientific or primitive, with little effort to investigate their underlying pharmacological potential or their social role in communities. Further, representations of Africa in global health imagery, for example, photographs of deprived children or rural poverty, often reinforce deficit narratives rather than resilience, innovation or agency.

This article argues that decolonising global health in Africa requires a constructive, multi‐layered shift, one that recentres African agency, values and resources; re‐examines the visual and discursive framing of African health; legitimises plural epistemologies, including indigenous and traditional systems; and fosters equitable partnerships in which Africans are not simply recipients but leaders, co‐creators and innovators. The remainder is structured as follows: (1) a brief historical legacy of coloniality in global health; (2) representation, imagery and the politics of narrative; (3) epistemic injustice and the marginalisation of African knowledge; (4) repositioning global health practice in Africa pathways and recommendations; and (5) conclusion. This article extends existing debates on decolonising global health by repositioning Africa not as a passive site of intervention but as an active locus of innovation, knowledge and resilience. Unlike much of the existing literature, which primarily critiques funding asymmetries and authorship imbalances, this article foregrounds the epistemic and representational dimensions of decolonisation, how African realities are portrayed, and how indigenous health practices are undervalued.

### 1.1 The Colonial Legacy in Global Health

The structures and logics of today's global health field cannot be understood without attending to their historical roots. Colonial administrations established health systems in Africa primarily to serve extraction, labour productivity and the interests of colonising powers, not necessarily the holistic health of local populations. This historical pattern has been well documented in African medical histories, which show that colonial health structures served economic extraction and labour protection rather than community health [9, 10].

With independence, many African health systems inherited the architecture, protocols and funding dependencies that were shaped under colonial or neo‐colonial logics. As Mulumba et al. [9] and Lawrence and Hirsch [10] argue, global health continues to reproduce entrenched imbalances through agenda‐setting concentrated in the global north and inequitable research partnerships and funding structures that limit African autonomy [9]. These power imbalances are currently manifested in multiple ways, including decision‐making and agenda‐setting disproportionately located in the Global North; research partnership models where African institutions are data‐gathering sites rather than leaders; funding flows that condition priorities; intellectual property regimes that limit technology transfer; and global value chains in pharmaceuticals and vaccines that keep African countries at the periphery [5, 11]. This reflects a broader centre–periphery logic rooted in coloniality, where knowledge, expertise and authority are concentrated at the ‘centre’ (typically global north institutions), whereas African institutions occupy an externally defined ‘periphery’ that is positioned as dependent, less authoritative or merely implementational [12].

**Figure:**
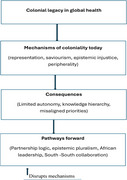
Conceptual framework for analysing the decolonisation of global health in Africa. The framework illustrates how colonial legacies in global health shape contemporary mechanisms of coloniality, which, in turn, generate systemic consequences. The pathways forward represent decolonising interventions that respond to and act back on these mechanisms and their consequences to enable more equitable and locally grounded global health practice. *Source:* From authors.

**BOX 1 | Definition of key concepts**

**Coloniality**: In this article, we define coloniality as the persistent patterns of power, knowledge and resource control that continue to exist beyond formal colonialism, influencing whose expertise is valued and whose priorities define the global health agenda [1].

**Saviourism**: In the context of this article, saviourism is defined as a paternalistic dynamic in which actors from the global north are positioned as rescuers of the Global South, often reinforcing racialised hierarchies and obscuring local agency, expertise and leadership [13].

**Representation inequity**: In this article, we define representation inequity as disproportionate visibility and narrative control by global north institutions in global health imagery, publications, policy framing and authorship, often marginalising African voices and perspectives [14, 15].

**Epistemic injustice**: Epistemic injustice is the systematic undervaluing, exclusion or dismissal of certain groups as legitimate knowers or producers of knowledge, often affecting Indigenous, African and community‐based knowledge systems in global health [16, 17].

**Epistemic pluralism**: We refer to epistemic pluralism in this article as the recognition that multiple knowledge systems, including biomedical science, traditional medicine, indigenous healing and community‐based practices, possess validity and should coexist within research, policy and practice [18].

**Partnership logic**: We define partnership logic as equitable collaboration, shared decision‐making and locally led governance, contrasting with donor‐recipient models that centralise power and agenda setting in the global north [19].

**South–South Collaboration**: In this article, South–South collaboration is understood as the cooperative knowledge exchange and capacity building among countries of the Global South, often including Africa, Latin America, the Caribbean and Asia, enabling shared decolonial strategies and mutual strengthening [18, 20].

In a recent review of Africa's quest of pharmaceutical sovereignty, Mulumba et al. [9] posit that Africa's continued reliance on imported medicines, vaccines and active pharmaceutical ingredients is the direct legacy of colonial extraction. This affirms that global health remains structured and regulated in a manner that the ‘centre’ defines for the ‘periphery’ rather than co‐creates with it. Therefore, decolonising global health is not merely rhetoric but must encompass power redistribution, shifting from a beneficiary model to an agent model, and enabling local leadership, capacity, production and regulation.

### 1.2 Representation and Politics of Imagery

One important but frequently under‐examined dimension is how global health uses imagery, narrative and communication to represent Africa. Photographs of malnourished children, rural clinics in ruin or people in dire need are ubiquitous in campaign materials, NGO websites and research reports. On the surface, these images evoke urgency and empathy, yet they also implicitly reinforce a narrative of African helplessness, deficiency and external salvation. Such imagery serves moral fundraising appeals, but it risks reducing Africans to passive objects of intervention rather than active subjects. Development and humanitarian scholarship demonstrate that these visual tropes reproduce racialised hierarchies, infantilise African subjects and legitimise paternalistic interventionism [14, 15]. They also align with the broader white saviour logic in which Western actors are positioned as rescuers of passive African victims, thereby entrenching unequal power relations between the provider and the receiver [13, 21]. A decolonised approach to global health communication would instead highlight African innovation, community agency, locally driven solutions and resilience. It would avoid using images just as ‘poverty porn’ or ‘victim pictures’ and instead portray dignity, capability and ownership. Additionally, representation in research and policy matters, such as who is quoted, who sets the frame, and whose voice is heard? If African scholars are under‐represented or overshadowed by foreign voices, the narrative remains skewed.

### 1.3 Epistemic Injustice and Marginalisation of Local Knowledge

Another key area is knowledge legitimacy. The global health discourse often privileges Western biomedical models, disregarding or dismissing traditional medicines, cultural practices of care, communal health systems and indigenous epistemologies. Yet, in many African settings, such practices are not only meaningful culturally but are also significant in terms of access, acceptability and outcomes. Failure to engage with these practices constitutes an epistemic injustice where the knowledge of local communities is undervalued or ignored. This aligns with Fricker's [8] argument that certain groups are systematically discredited as knowers and with Ndlovu‐Gatsheni's [7] account of how coloniality continues to delegitimise African knowledge. This not only disrespects the cultural logic of communities but also misses opportunities for innovation and culturally congruent interventions.

As noted by Tuck et al. [22], to overhaul the colonial base of global health, a paradigm shift is a keyway in which this could be achieved. The shift required is from epistemic injustice towards epistemic pluralism, a recognition that multiple knowledge systems, including traditional medical systems, have legitimacy and can coexist within health research and policymaking. In practice, this means that global health research, evaluation and interventions should go beyond asking, ‘Does a Western‐designed intervention work in Africa?’ to ‘What local practices, cultural logics and community resources can be mobilised, adapted or scaled to improve health outcomes?’ and do so with respect, co‐design and without automatic dismissal. It also means interrogating claims that cultural practices or herbal medicines are inherently risky without contextualised, evidence‐based investigation. Instead of simply labelling them as unscientific, researchers should engage in rigorous study, respect epistemic diversity and open avenues for integration or respectful pluralism.

### 1.4 Repositioning Global Health in Africa: Pathways and Recommendations

African scholars, practitioners and policymakers must lead the design, implementation and evaluation of health programmes [2]. Too often they are involved only as local implementing partners. Research partnerships should be equitable where African institutions should co‐design agendas, lead authorship, hold budgets and exercise governance. Ssennyonjo et al. [23] emphasise that Africans must ‘self‐decolonise’ by acknowledging local weaknesses and forging home‐grown solutions aligned with continental frameworks such as Agenda 2063.

Again, health interventions should be co‐created with communities, leveraging local knowledge and organisational resources. They should build on existing cultural practices of care rather than replacing them wholesale. For instance, many African community health worker models, traditional healers’ networks or communal care systems may offer innovation. Partnerships should aim to adapt, integrate and strengthen these rather than ignoring them. It is further recommended that global health research explicitly acknowledge and value multiple epistemologies that involve respectful engagement with traditional medicine, communal healing practices and cultural health logics [24]. This aligns with broader calls for equitable partnerships, fair authorship and shifting funding power to LMIC institutions [25, 26]. Research ethics and design should be sensitive to indigenous realities, avoid imposing Western frameworks uncritically and question whose knowledge counts. This transformative paradigm proposed in the literature suggests research should respond to social and health injustice and be rooted in values of the communities impacted [2].

Moreover, a critical element of decolonising global health in Africa is enabling pharmaceutical and health‐technology sovereignty. The recent review on Africa's pharmaceutical sovereignty highlights how structural barriers (patents, regulatory weakness, and underinvestment) prevent local production and maintain dependency [2, 27]. Building local manufacturing capacity for medicines, diagnostics and vaccines, strengthening regulatory agencies and pooling regional procurement are all key strategies. These shift Africa from being a passive site of implementation to a hub of innovation, manufacturing and regulation. Additionally, global health organisations and journals should adopt ethical frameworks for how Africa and Africans are represented. This means avoiding stereotypical ‘poverty’ images and instead showcasing agency, innovation and solutions‐led narratives. It means including local voices, authors and community images that reflect dignity and ownership rather than purely deficit. Discourse around culture, herbal medicine or community practices should be nuanced, evidence‐based and respectful rather than dismissive.

Furthermore, global health funders need to shift from a donor and recipient logic to a partnership logic. This shift will create an enabling environment in which African institutions can access funding directly, exercise greater decision‐making autonomy and build sustainable capacity rather than perpetuating dependency. Research collaborations should also ensure fair authorship, equitable local governance, data ownership and open access. Ultimately, strengthening African institutions, including universities, regulatory bodies and public health agencies, is foundational. Ssennyonjo et al. [23] argue that strong institutions are essential for driving multilevel, collaborative action at scale and that the decolonisation agenda aligns with Africa's own strategic aspirations, such as Agenda 2063 and the Sustainable Development Goals (SDGs). Again, although this article focuses on Africa, the wider movement to decolonise global health is strengthened through South–South collaboration. Across Latin America, the Caribbean and other regions of the Global South, Indigenous and decolonial scholars have articulated parallel critiques of coloniality and the unequal valuation of knowledge systems. Foundational contributions from Latin American thinkers, such as Quijano [12] and Walsh [16], have offered insightful conceptual tools for interrogating the universality of Western epistemologies and for advancing epistemic justice. Building cross‐regional alliances and co‐production networks between African and other Global South actors offers opportunities for shared learning, mutual support and the development of pluralistic, contextually grounded approaches to global health.

## 2 Conclusion

Decolonising global health in Africa is not about rejecting Western medicine or isolating Africa from global knowledge. Rather, it is about recalibrating power, expanding what counts as valid knowledge, repositioning Africans as agents rather than objects and building systems that reflect the realities, cultures and resources of African communities. Such a transformation demands honesty about colonial legacies, courage to shift institutional practices and commitment to equity, dignity and agency.

Embracing indigenous leadership, co‐creation, knowledge pluralism, production sovereignty and ethical representation, global health in Africa can evolve from a paradigm of external aid dependency to one of innovation, partnership and self‐determination. In doing so, Africa can not only solve its health challenges but also contribute knowledge, technologies and models to the global health ecosystem, reversing the one‐way flow of knowledge and interventions and embracing a genuinely globalised yet locally grounded health paradigm.

## Author Contributions

Conceptualisation: Michael Sarfo. Methodology: Michael Sarfo. Formal Analysis: Michael Sarfo. Writing (initial draft): Michael Sarfo and Ruth Owusuaa. Writing (revision and editing): Michael Sarfo and Ruth Owusuaa. Supervision: Michael Sarfo.

## Funding

Authors received no funding for this study.

## Consent

The authors have nothing to report.

## Conflicts of Interest

The authors declare no conflicts of interest.

## Data Availability

The study did not use any data.
